# Altered Plasma Mitochondrial Metabolites in Persistently Symptomatic Individuals after a GBCA-Assisted MRI

**DOI:** 10.3390/toxics10020056

**Published:** 2022-01-26

**Authors:** DeAunne Denmark, Ilene Ruhoy, Bryan Wittmann, Haleh Ashki, Lorrin M. Koran

**Affiliations:** 1 Department of Behavioral Neuroscience, Oregon Health & Science University, 3710 SW US Veterans Hospital Road, Mail Code R&D40, Portland, OR 97239, USA; denmarkd@ohsu.edu; 2 Mount Sinai South Nassau Chiari-EDS Center, 1420 Broadway, Hewlett, NY 11557, USA; iruhoy@drruhoy.com; 3 Owlstone Medical, 600 Park Offices Drive, Suite 140, Research Triangle Park, NC 27709, USA; Bryan.Wittmann@owlstone.co.uk; 4 Prime Genomics, Inc., 319 Bernardo Avenue, Mountain View, CA 94041, USA; halehashki@gmail.com; 5 Department of Psychiatry and Behavioral Sciences, OCD Clinic, Stanford University Medical Center, 401 Quarry Road, Stanford, CA 94305, USA

**Keywords:** gadolinium-based contrast agents (GBCAs), gadolinium, mitochondrial disease, metabolomics, oxidative stress

## Abstract

Despite the impressive safety of gadolinium (Gd)-based contrast agents (GBCAs), a small number of patients report the onset of new, severe, ongoing symptoms after even a single exposure—a syndrome termed Gadolinium Deposition Disease (GDD). Mitochondrial dysfunction and oxidative stress have been repeatedly implicated by animal and in vitro studies as mechanisms of Gd/GBCA-related toxicity, and as pathogenic in other diseases with similarities in presentation. Here, we aimed to molecularly characterize and explore potential metabolic associations with GDD symptoms. Detailed clinical phenotypes were systematically obtained for a small cohort of individuals (*n* = 15) with persistent symptoms attributed to a GBCA-enhanced MRI and consistent with provisional diagnostic criteria for GDD. Global untargeted mass spectroscopy-based metabolomics analyses were performed on plasma samples and examined for relevance with both single marker and pathways approaches. In addition to GDD criteria, frequently reported symptoms resembled those of patients with known mitochondrial-related diseases. Plasma differences compared to a healthy, asymptomatic reference cohort were suggested for 45 of 813 biochemicals. A notable proportion of these are associated with mitochondrial function and related disorders, including nucleotide and energy superpathways, which were over-represented. Although early evidence, coincident clinical and biochemical indications of potential mitochondrial involvement in GDD are remarkable in light of preclinical models showing adverse Gd/GBCA effects on multiple aspects of mitochondrial function. Further research on the potential contributory role of these markers and pathways in persistent symptoms attributed to GBCA exposure is recommended.

## 1. Introduction

With excellent general safety profiles, gadolinium (Gd)-based contrast agents (GBCAs) are a mainstay for medical imaging. Chelator conjugation facilitates typically rapid renal excretion, averting the ordinarily high biotoxicity of free ionic Gd due to Ca^2+^ size similarity and interference with critical physiological processes [1,2]. Safety exceptions emerged initially with the discovery of Nephrogenic Systemic Fibrosis (NSF) in patients with pre-existing renal compromise [2]. Despite the eradication of new cases, pathological mechanism(s) remain incompletely understood, and new toxicity concerns are arising from evidence of Gd retention, particularly in the brain and bone [2], despite normal renal function.

Gd-induced necrosis and apoptosis of diverse cell types and fibrotic chemokine/cytokine activity are reported in various preclinical models [3]; however, no clinical histological evidence or relationship between GBCA dosing and neurological outcomes has been reported [3]. Nonetheless, plausible health risks prompted the European Medicines Agency to recommend the discontinuation of several GBCAs [2], and GDD was proposed as a new clinical entity following increasing reports of severe, persistent, post-exposure symptoms concomitant with ongoing Gd excretion [3,4]. 

Impaired mitochondrial function and an elevated reactive oxygen species (ROS) are emerging as mechanistic themes of toxicity. Gd^3+^ decreases membrane potential (Ψ_m_) and increases fluidity, cytochrome c (cyt-c) release, and ROS in whole cells and isolated liver mitochondria, suggesting cell entry and direct binding [5,6]. GdCl_3_-induced apoptosis of rat cortical neurons was accompanied by time- and dose-dependent ROS increases, decreased ATP, Ψ_m_ depolarization, cyt-c release, and caspase-3 activation [7]. Similar effects were observed in other cell types [8], even after low-dose macrocyclic agents [9], including cell death and mitochondrial toxicity in human neurons representing basal ganglia [10], the region of highest clinical deposition [11]. Recently, neuron-derived extracellular vesicles (NDEVs) from GDD patient plasma showed altered amounts of specific mitochondrial proteins vs. controls [12]. Antioxidant pretreatment prevented neuronal Gd-induced cell death and ROS-associated endoplasmic reticulum stress, and reduced both GdCl_3_ and macrocyclic GBCA-associated oxidative stress and toxicity in chronic renal failure models [13]. 

As a result of the resemblance between GBCA-attributed symptoms and those common in mitochondrial diseases (MDs) [14,15], we hypothesized involvement of similar pathophysiology. Substantial clinical and etiological heterogeneity renders MDs notoriously difficult to diagnose [15,16]; a spectrum of diverse metabolic consequences can arise from the shared fundamental feature of compromised oxidative phosphorylation (OXPHOS). Discovery-based untargeted metabolomics approaches profile hundreds or more unique small molecules in a single sample to relate biochemical mechanisms to cellular or physiologic phenotype [17,18], both distinguishing primary MDs [19,20,21] and improving characterization of conditions involving secondary dysfunction, e.g., Type 2 diabetes (T2D) [22]. Here, we took advantage of these strengths in combination with detailed clinical assessment to explore whether persistently symptomatic GBCA-exposed individuals show plasma alterations that suggest mitochondrial and/or other metabolic contributions. 

## 2. Materials and Methods

Clinical assessment. Clinical and biological data from adult participants (>18 years; n = 11 female, 4 male) were utilized under a Stanford IRB-approved consent [23]. All met provisional GDD diagnostic criteria: (1) new onset (≤30 days from GBCA-assisted MRI) of ≥3 symptoms: cognitive difficulty; pain in extremity, chest wall, skin, or joint (arthralgia); frequent headaches; skin thickening and/or hyperpigmentation; and, (2) unprovoked 24h urine Gd amount exceeding the laboratory reference limit ≥28 days post-MRI. Plasma samples were obtained ≤3 years of the symptom-associated MRI. 

At blood draw, patients completed a rating questionnaire for GDD diagnostic symptoms and others commonly reported [3,4]: tingling (paresthesia), muscle twitching and/or pain, fatigue, decreased sensation (skin, bowel, or bladder), bone pain, decreased visual acuity, and eye pain and/or dryness. Depending on the numerical rating scale, symptoms were categorized as severe (8–10/3–4), moderate (5–7/2–3), and mild (1–4/1–2). Zero (0) and blanks were considered absent and omitted. Scores were calculated for total symptoms (maximum = 109) and MD-like symptoms (maximum = 67) per previous reports [14,15,24]. 

Plasma metabolomics. Whole blood samples were collected (*n* = 10 fasting 8–10 h) by antecubital venipuncture, and plasma separated, aliquoted (1.0 mL), and frozen immediately (−80 °C). Extraction and untargeted metabolomics analyses were conducted by Metabolon, Inc. (Durham, NC, USA) using four independent, ultra-high performance liquid chromatography tandem mass spectrometry (UPLC-MS/MS) platforms run in parallel and covering analytes across 70–1000 *m/z*, as detailed previously [25]. Raw data were extracted, peaks identified, and compounds confirmed with an internal library of >4500 purified standards based on retention time/index, *m/z*, and chromatographic data. Rigorous quality control and curation ensured accuracy and consistency, and removed system artifacts, misassignments, and background noise. 

Data analysis and statistics. Raw biochemical values correspond to integrated intensity values as calculated using the area under the chromatographic peak. A semi-quantitative analysis was achieved by comparing participant samples to a set of invariant anchor samples (matrix) included in each run batch, as previously outlined [26]. A stepwise relative quantitation approach similar to clinical laboratory analyses was used to identify metabolites for which participant values were more extreme (altered) than in reference samples. Raw spectral intensities for each biochemical in participant samples were normalized to anchor samples, log transformed, and compared to plasma samples of a reference cohort of self-reported healthy, asymptomatic, non-fasting adults (n = 34 female, n = 19 male) enrolled as controls in a previous IRB-approved study (unpublished). Output values for each biochemical were generated as Z-scores—a semi-quantitative measurement of a value’s relationship to a comparator group mean and standard deviation (SD). Here, participant Z-scores for a given biochemical represent the number of SDs from the normalized mean signal intensity of that biochemical in the reference cohort. Data showed consistent log-normality, and natural log-transformation was applied to each biochemical detected. Reference intervals (RI) were set based on the specific distribution of normalized signal intensities for the reference cohort, approximating the span between the lower (<2.5th percentile) and upper (>97.5th percentile) tails, i.e., ±~2 SDs the median-scaled log-transformed mean. 

Biochemicals detected in ≥24% of the reference cohort were included in all analyses. Where values were missing, only high-tail RI values were reported and used to determine participant altered status. Analytes detected in <50% of participant samples were excluded, though flagged for potential relevance if the RI was two-tailed. Among the analytes remaining, those for which ≥60% of participant Z-scores fell ~1 SD above (85th percentile) or below (15th percentile) the reference Z-score mean were considered altered. Values outside the RI (±~2 SDs) were also noted, as were the number of patients with altered Z-scores in each quantile, and in total per analyte. If the RI included a low tail, absent Z-scores were considered below the lower limit of detection, altered, and assigned a minimum value just below either the lowest participant Z-score or the RI low tail. For analytes with only high tail reference values, missing individual participant Z-scores were not imputed and were excluded from group analyses. Median participant Z-scores were calculated for all analytes considered altered as above.

Multivariate analyses with hierarchical clustering and Principal Components Analysis (PCA) were performed in R (version 3.6.1, R Foundation for Statistical Computing, https://www.R-project.org, accessed on 26 January 2021) on the initial set of all biochemicals passing inclusion filters to explore for any underlying structure, including that related to age, sex, total symptom score (high/low vs. median), and urine Gd levels (high/low vs. median). Pearson correlation and standard linear regression analyses were used to evaluate associations between urine Gd levels, days from symptom-associated MRI to urine Gd sampling and blood draw, lifetime number of MRIs, symptom scores, and biochemicals altered in patients (*p*-value < 0.05).

## 3. Results

### 3.1. Participant Clinical Characteristics

Median age at blood draw was 50 years (range 28–82 years). As shown in Table 1, symptoms began within 72 h for all but one participant, and were attributed to both macrocyclic (*n* = 10) and linear (*n* = 5) GBCAs. The lifetime number of GBCA-assisted MRIs varied (1, *n* = 8; 2, *n* = 3; 3, *n* = 1; 4, *n* = 1; ≥9, *n* = 2). Consistent with recent evidence that GBCA excretion rates may be significantly lower than originally estimated in individuals with apparently normal renal function [27], patient urinary Gd at 1–7 months post-MRI ranged from 1.0 µg to 33.0 µg, well above clinical laboratory thresholds for unexposed individuals. Time from initial symptom-attributed MRI was only moderately negatively associated with urine Gd (log–log R^2^ = −0.478; *p* = 0.0043), suggesting additional influences on excretion may be present.

Symptom frequency at blood draw is shown in Table 2. Most are considered clinically relevant criteria for suspicion of MD [14,15,16], with the five most prevalent regularly being patient self-reported [24]. As shown in Table 1, total severity was driven predominantly by MD-like symptoms (*r* = 0.97, *p* < 0.001). The shortest time from symptom onset to blood draw was 70 days (mean = 330 days; median = 248 days), suggesting an ongoing and potentially progressive process in some cases. Congruent with reports that GDD symptom chronicity is independent of cumulative GBCA dose and retention [23,28], no association was detected between urinary Gd and total or MD symptom scores (*r* = −0.12, *p* = 0.67; *r* = −0.19, *p* = 0.50), nor did severity correlate with days between the symptom-related MRI and symptom assessment (total: *r* = −0.06, *p* = 0.83; MD: *r* = −0.01, *p* = 0.97). However, a moderately significant association was seen between number of lifetime MRIs and MD symptom score (*r* = 0.53, *p* = 0.04), with a near-significant trend for total symptom score (*r* = 0.47, *p* = 0.08). 

To minimize potential confounding of subsequent metabolomics results, we assessed patients for the presence of concurrent medical conditions and/or associated treatments. At blood draw, eight patients had one or more of eight active, distinct medical diagnoses for which existing studies suggest mitochondrial involvement: irritable bowel syndrome (*N* = 4), “anxiety” or generalized anxiety disorder (*N* = 2), hypothyroidism (*N* = 2), hypertension (*N* = 2), hyperthyroidism (*N* = 1), mast cell activation syndrome (*N* = 1), fibromyalgia (*N* = 1), and depression (*N* = 1). Seven of these patients plus two others were taking one or more medications (amlodipine, aspirin, benzodiazepine, beta-blocker, diphenhydramine, duloxetine, ergoloid mesolate, N-acetylcysteine, sertraline, thyroid hormone) and/or supplements (ashwaganda, astaxanthin, coenzyme Q10, glutathione, vitamin C) with evidence supporting a role in preserving or aiding mitochondrial function. Given such heterogeneity, any consistent effect(s) and associated bias on plasma metabolomic profiles is unlikely.

### 3.2. Metabolite Alterations in Symptomatic GBCA Exposure

**Figure 1:**
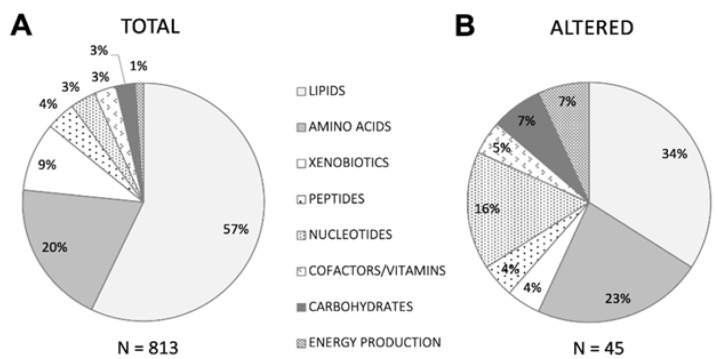
Pie chart depicting the proportion of biochemicals belonging to each of eight major metabolic superpathways detected in (**A**) the total passing filtering criteria among participants and reference cohort individuals, and (**B**) the subset meeting altered criteria among participants compared to the reference cohort, as described in Methods.

As immediate reflections of cellular and physiological status are not subject to epigenetic or post-translational modification, small molecule metabolites provide a functional readout closely correlated with phenotype [17,18]. Untargeted mass spectrometry measures molecular masses to determine identity and provides high sensitivity, throughput, and versatility to elucidate complex analyte mixtures, enabling discovery-based molecular characterization of both health and disease [29]. Using this approach, we identified 960 unique biochemicals in plasma from GBCA-exposed and reference cohorts; 813 across eight metabolic superpathways passed quality filters (Materials and Methods) and were included in further analyses (Figure 1). 

Figure 1 Superpathways represented by biochemicals detected in plasma metabolomics analyses.

Interindividual variability of peripheral metabolite levels attributable to factors such as age, gender, fasting status, and genetics [17,25,29] appears to be small overall but statistically significant [30]. Two-way hierarchical clustering and unsupervised multivariate analysis with three-dimensional PCA on the filtered set of biochemical Z-scores from all patients revealed no obvious patterns (data not shown); dispersed heterogeneity among biochemical profiles indicated no apparent contribution from these covariates toward any underlying structure. Neither clustering nor PCA distribution was associated with age, gender, or other clinical variables (urine Gd, total and MD symptom scores), suggesting interference from these factors was unlikely.

**Figure 2:**
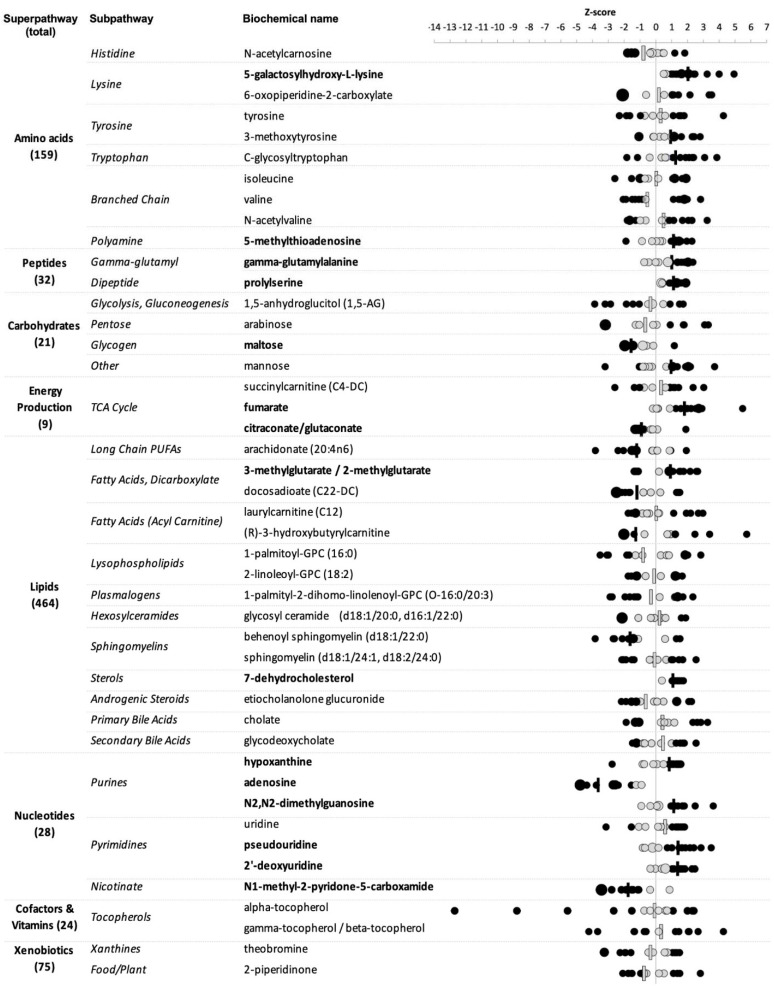
Specific biochemicals and their respective pathways identified as potentially altered in GBCA-exposed patients. Circles indicate individual patient Z-scores with larger sizes denoting multiple patients; bars indicate median values; black—values 1 SD above or below the normalized reference cohort mean, gray—values less than 1 SD from the normalized reference cohort mean. Bolded biochemical names indicate those for which alterations were directionally consistent across patients.

To enable detection of potential symptom-relevant perturbations, we used a stepwise relative quantitation approach similar to clinical laboratory testing, which emphasizes comparisons between individual values and the range (RI) associated with a defined reference cohort over averaged group differences. Combining relative quantitation with pathway analysis, which favors identification of relevant biologic processes rather than single biomarkers, including those for which bi-directional alterations may be phenotypically relevant and is more tolerant of population heterogeneity [31], helps leverage the systems-based strengths of metabolomics for meaningful use in clinical samples. For each of the 813 biochemicals, we designated as provisionally altered those for which at least nine participant Z-scores were ±~1 SD (>85th or <15th percentile) the normalized reference cohort mean, also noting how many fell outside the more stringent ±~2 SDs (>97.5th or <2.5th percentile) thresholds. This criterion was met by 45 biochemicals (Figure 2), with patient Z-scores higher than the reference mean for 20, lower for 15, and split between high and low for ten. All eight metabolic superpathways were represented, with the proportions of nucleotides (15.9%) and energy production (6.8%) notably greater—4.6 and 6.1 times greater, respectively—than their proportions (3.5% and 1.1%) in the total set identified (Figure 1).

Figure 2 Altered metabolites in GBCA-exposed patients with persistent symptoms.

We first assessed this subset for group-level perturbations. Median patient Z-scores surpassed 1 SD for 20/45 biochemicals (solid black dashes in Figure 2), including two >2 SDs above (5-galactosylhydroxy-L-lysine) or below (adenosine) the normalized reference cohort mean. Of the remaining, 13 were above and 7 below. Nucleotide superpathway over-representation was again notable; 6/7 biochemicals with this functional designation appeared altered compared to the reference.

Given the small and heterogeneous cohort, we hypothesized that examining individualized altered metabolite patterns might be informative. For 12/45 biochemicals, the direction of altered individual Z-scores was entirely consistent—10 high (5-galactosylhydroxy-L-lysine, 5-methylthioadenosine, gamma-glutamylalanine, prolylserine, fumarate, 3-methylglutarate/2-methylglutarate, 7-dehydrocholesterol, *N*2,*N*2-dimethylguanosine, pseudouridine, 2′-deoxyuridine) and 2 low (adenosine, *N*1-methyl-2-pyridone-5-carboxamide). Three biochemicals showed directional consistency in all but one participant (high—hypoxanthine; low—maltose; citraconate/glutaconate). Median patient Z-scores were also outside 1 SD of the RI mean for all 15 of these biochemicals (names bolded in Figure 2), including all nucleotides and 2/3 energy production metabolites, further suggesting possible contributory roles and promise as candidates for further investigation. 

For the remaining 30 metabolites, individual Z-scores fell both above and below 1 SD of the RI mean Figure 2), with 20 either high or low in the majority of patients. In contrast to those showing complete directional consistency, Z-scores were predominantly low (12), not high (8), for biochemicals in this mixed group. Altered individual Z-scores were split (~50%) in both high and low directions for the remaining 10. All altered lipids were among these mixed groups, as were others known to associate with food intake (e.g., 1,5-AG, theobromine), which may relate in part to obtaining plasma samples from patients both fasting and not.

Finally, we explored relationships between altered biochemicals and clinical variables. To select for the most meaningful, we set a threshold for the absolute value of correlation coefficients (|*r*|) to |*r*| > 0.8. No altered biochemical Z-scores were associated with urine Gd excretion levels, symptom scores or days from MRI to blood draw (data not shown). However, six highly significant metabolite–metabolite correlations were identified: isoleucine-valine (*r* = 0.91, *p* < 0.0001), sphingomyelin (d18:1/24:1, d18:2/24:0)-arabinose (*r* = 0.90, *p* < 0.0001), pseudouridine-*N*2,*N*2-dimethylguanosine (*r* = 0.90, *p* < 0.0001), C-glycosyltryptophan-pseudouridine (*r* = 0.87, *p* < 0.0001), C-glycosyltryptophan-*N*2,*N*2-dimethylguanosine (*r* = 0.85, *p* < 0.0001), and fumarate-1-palmityl-2-dihomo-linolenoyl-GPC (O-16:0/20:3) (*r* = -0.80, *p* < 0.005). Remarkably, half of these were biochemicals from the nucleotide superpathway.

## 4. Discussion

This preliminary plasma metabolomics study of individuals with persistent MD-like symptoms arising after GBCA exposure presents small molecule evidence of a possible contributory role for mitochondrial dysfunction. MDs are renowned for extensive clinical and genotypic variability and highly diverse disease presentations related to consequences of OXPHOS deficits, e.g., excessive ROS, intermediary metabolite accumulation, and decreased ATP [32]. While primary MDs typically result from mitochondrial and/or nuclear DNA mutations [14,15], secondary MDs are increasingly recognized in association with environmental exposures, infection, and chronic non-communicable disease, e.g., T2D [22]. Frequent nonspecific neuromuscular and other symptoms, and fluctuating and/or progressive symptomatology, make MD diagnosis particularly challenging in adults [14,16].

Pain and paresthesia were among the most common GBCA-attributed symptoms here and previously [3,4], and may represent peripheral neuropathy, a regular and defining feature in some MDs that can lack histological or biochemical evidence [33]. Similarly, headache and migraine are increasingly appreciated as prevalent in MDs [24,34], as are the central roles of mitochondrial dysfunction and oxidative stress in migraine etiology [34]. Gd deposition has recently been linked with fibromyalgia, a poorly understood entity clinically overlapping GDD with chronic neuropathic/myopathic pain, headache, and cognitive difficulty [35], and in which ROS-related damage, aberrant mitochondria morphology, reduced respiratory activity, and a distinct metabolomic profile have been observed [36]. 

Definitive MD diagnosis usually requires biopsy and/or genetic testing; no specific metabolic markers can reliably identify a particular defect, and while conventional urine or plasma abnormalities sometimes correlate, their diagnostic sensitivity and specificity remain poor [15,16]. Small molecules are increasingly globally interrogated in clinically heterogeneous conditions involving mitochondria, e.g., inborn errors of metabolism (IEMs) [37], and to characterize and differentiate primary MDs [19,20], thereby greatly expanding biomarker identification. Our discovery-based MS metabolomics analyses identified 45 plasma biochemicals as provisionally altered in this small GDD cohort, with the proportion of plasma biochemicals from nucleotide and energy superpathways notably higher than expected. Additional alterations in specific amino acids and their derivatives, in lipids, and in oxidative stress and other markers were also observed, consistent with multiple abnormalities reported in both primary and secondary MDs.

Nucleotides. Several purines, pyrimidines, and nicotinate (NA) derivatives were altered across patients, ~5× more than expected for this superpathway, and directionally consistent in almost all cases. Nucleotide pathways have been repeatedly implicated in MDs, including in limited human studies [19,20]. *N*1-methyl-2-pyridone-5-carboxamide, a pyridine derivative and main nicotinamide (NAM) metabolite whose precursor NA (niacin; vitamin B_3_) is critical for adequate NAD+ and redox balance maintenance, was uniformly low. NAM Z-scores also trended toward, but did not reach, our lower significance thresholds in most cases, suggesting potential demand in excess of supply. Major NAM coenzymes, e.g., NAD+/NADH, are challenging to measure in vivo and were not assessed here, but may be of great interest. Considering that all NA precursors can enter human cells to support mitochondrial NAD+ generation and potentiate function [38] and appear to be safe nutritional supplements, this pathway may also be a viable therapeutic target. 

*N*2,*N*2-dimethylguanosine and pseudouridine, common transfer RNA (tRNA) modified nucleosides crucial for stable and accurate translation, were elevated in most patients and highly correlated. The tRNA pool is dynamically regulated to facilitate appropriate and timely protein expression, especially in response to stressors such as ROS. Mutations in tRNA genes, and in those for tRNA processing, charging, and modification enzymes, are causal for the most common primary MDs [39]. Modified nucleosides are also associated with diverse diseases involving secondary mitochondrial dysfunction, e.g., T2D, cancer, and viral infection [39].

Energy production. Elevated lactate is a well-known perturbation in MD, and blood lactate–pyruvate ratio (L:P) in this setting reliably differentiates MRC and pyruvate metabolism disorders, though with considerably low sensitivity [19]. Lactate is normal in many MD patients, or elevated only following exercise or metabolic crisis. While neither pyruvate nor lactate were altered in our cohort, two TCA cycle intermediates (fumarate and citraconate/glutaconate) were elevated and reduced, respectively. Since high NADH inhibits three TCA cycle dehydrogenases, MRC impairment with a lowered NAD+–NADH ratio may lead to congestion and altered intermediates in plasma, urine, or both, as reported in some MD metabolomics studies [20]. Three carnitine species were also altered, including 3-hydroxybutyrylcarnitine, interpreted as hindered mitochondrial fatty acid beta-oxidation in primary MD [40]. Significant persistently deranged carbohydrate and energy metabolism were also seen by NMR metabolomics in rat models of GBCA exposure [41].

Amino acids. Branched-chain amino acids (BCAA) isoleucine, valine, and *N*-acetylvaline, were altered in most patients, along with keto acid derivatives in some, consistent with compromised catabolism resulting from MRC dysfunction and associated redox imbalances in both primary [40] and secondary [22] MDs. BCAA and glucose metabolism are tightly interconnected; as such, altered carbohydrates (maltose, arabinose, and mannose) and 1,5-AG, a deoxyhexose reflecting postprandial hyperglycemia and sensitive marker of short-term glycemic control, further suggest potential metabolic issues similar to primary MDs [15,16]. While all other amino acids appeared similar to the reference cohort, altered tyrosine and 3-methoxytyrosine (3-*O*-methyldopa) in a majority of (and the same) patients may suggest possible relevance of monoamine pathway(s), which are renowned for central roles in pathogenic mitochondrial ROS generation in neurodegenerative and other diseases [42]. 

5-(galactosylhydroxy)-L-lysine is a bone matrix type I collagen terminal degradation product released into bloodstream during resorption; increased urinary concentrations are a validated marker in metabolic diseases [43]. Considering that bone is a prominent reservoir for Gd deposition, and the good correlation between bone and brain levels [44], the uniformly high and most severe alternation of this biochemical among all examined is compelling. Pre-existing rapid bone turnover may be a GDD risk factor; more Gd could not only be taken up during active resorption and remodeling, but may also be freed by transmetallation and continually released into the circulation. Indeed, bone Gd levels >800-fold higher were observed in GBCA-exposed vs. naïve patients, and remained elevated for over eight years [44].

Lipids. The branched organic acid and leucine degradation product 3-methylglutarate/2-methylglutarate (3-MGA) was altered in almost all patients. Elevated urinary 3-MGA is frequently seen in IEMs, including organic acidurias, disorders of glycogen storage, fatty acid oxidation, and the urea cycle, and genetically proven primary MDs [45]. Leucine degradation and sterol biosynthesis are linked through the mevalonate shunt, which increases toward 3-MGA production when gluconeogenesis and cholesterol synthesis homeostasis is disturbed by NADP/NADPH-dependent enzyme compromise. Interestingly, the cholesterol precursor 7-dehydrocholesterol (7-DHC) was also uniformly high in our cohort. Low plasma cholesterol, with elevated 7-DHC and 3-MGA has been observed in the congenital metabolic disorder Smith–Lemli–Opitz Syndrome [46]. 

Oxidative stress. While not independent diagnostic parameters, markers of oxidative balance can provide important information about MD pathophysiology, continued vulnerability, and potential antioxidant therapeutic efficacy [47]. Oxidative stress compounds, including the purine derivative and tissue hypoxia/ROS marker hypoxanthine, and vitamin E isoforms α-, β-, and γ-Tocopherols (T), were altered in almost all patients. At least one T isoform was altered in all but one, with some low levels being among the most dramatic alterations overall. α-T, the primary tissue and supplement isoform, is well-known for potent ROS scavenging properties, and low plasma T, independent of dietary intake has been repeatedly, albeit inconsistently, considered a promising therapeutic target in some diseases related to mitochondrial ROS, e.g., Alzheimer’s [48]. The common antioxidant N-acetylcysteine protected rats from Gd-DTPA-induced injury [13] and reversed some associated plasma oxidative stress markers [13].

Cytokines and inflammation. Low GdCl_3_ and GBCA concentrations induced cytokine release in mouse macrophages, including TNF-α, IL-6, and IL-10, increased ROS, and suppressed Ψ_m_ [4]. As our cohort was a subset of GDD patients with higher peripheral TNF-α and IL-6 in association with new onset frequent headaches and neuropathic pain [23], the consistently elevated 5-methylthioadenosine (MTA), decreased adenosine, and altered arachidonic acid (AA) we observed may be clinically meaningful. MTA is an increasingly appreciated metabolic regulator of susceptibility and severity in sepsis; high plasma levels correlate strongly and positively with IL-6 and IL-8, and weakly with TNF-α, and signal a worse prognosis due to hyperinflammation [49]. Adenosine has potent immunomodulatory activity via widely distributed receptors that generally limit inflammation and support repair, but can contribute to immune suppression and fibrotic remodeling under sustained signaling [50]. The omega-6 polyunsaturated fatty acid AA is a primary precursor to eicosanoid lipid mediators, e.g., prostaglandins and leukotrienes, with central roles in pro- and anti-inflammatory signaling cascades, most of which are highly interconnected with cytokine dynamics. All of these play pivotal roles in immunometabolism, whereby immune cell activity, particularly macrophage and differentiating T-cell subsets, is under mitochondrial-mediated metabolic control [51]. 

This study is limited by small sample size, lack of case–control or prospective design, caveats inherent to metabolomics [30] and single platforms, and variability in sample timing. Additionally, some reference cohort individuals may have previously undergone GBCA-assisted MRI(s) despite self-reporting as healthy and asymptomatic. Whether variability in GBCA class or timing between receiving multiple doses induces different symptoms or severity should be investigated in larger GDD cohorts. Utilization of pain and other symptom rating scales along with other biophysical measures (e.g., electrophysiology) will be critical for more specific phenotyping, as will multi-modal molecular measures to confirm mitochondrial involvement (e.g., functional assays) and directly assess associated effects (e.g., oxidative stress). Our clinical laboratory approach does not guarantee statistical significance; however, incorporating anchor samples and log-transformation allows meaningful, exploratory, semi-quantitative assessment of both single biomarkers and functional metabolic pathways, and identified several highly plausible signals in this study. Biochemical alterations are not uniform even among genetically confirmed primary MDs [19,20,40], underscoring the phenotypic complexity of OXPHOS- and mitochondrial-related conditions, and the importance of leveraging methods that can resolve relevant individual variation.

## 5. Conclusions

Although preliminary, this study’s indications of potential mitochondrial involvement in GDD are notable both clinically and biochemically in light of preclinical studies showing adverse Gd/GBCA effects on multiple aspects of mitochondrial function. While persistent symptoms after GBCA exposure are rare, additional epidemiological and mechanistic research is needed, as are studies to identify preventive and therapeutic approaches. As in other GDD studies [4,23,28], symptoms often began after only one MRI. Together with observations that symptoms also did not occur until after two or more MRIs in many patients [4,23,28], this suggests that a cumulative dose is not critical to provocation if Gd is ultimately proven to be the symptom-provoking agent. Certain conditions (e.g., inflammatory diseases, high bone turnover) may represent risk factors requiring screening precautions similar to those for renal compromise [2]. Genetic factors and concomitant medications also merit investigation. 

## Figures and Tables

**Table 1:** Participant clinical and laboratory characteristics.

ID#	GBCA ^a^	MRI to Symptom Onset (Days)	Number of Lifetime MRIs	Total Symptom Score (max = 109)	MD Symptom Score (max = 67)	MRI to Urine Gd (Days)	24 h Urine Gd Level (µg)	MRI to Blood Draw (Days)
15	MV, MH, OM, G	0	32	85	57	127	1.9 ^b^	535
18	D	0	1	84	52	38	3.0 ^b^	293
8	MH, G	3	9	79	50	214	1.7 ^b^	248
6	G	3	1	74	48	89	1.0 ^b^	179
10	G	14	1	71	46	57	8.8 ^d^	265
11	OM	2	1	59	29	90	5.0 ^b^	695
7	G, MH	1	3	53	31	28	33.0 ^b^	71
1	G	3	1	47	28	41	26.0 ^c^	565
16	G	2	4	38	24	34	8.3 ^c^	128
4	G	1	2	38	24	87	1.3 ^b^	154
3	G, OP	0	1	36	21	36	22.0 ^b^	84
2	G	0	1	29	19	105	1.8 ^b^	450
9	G	1	1	28	22	77	3.9 ^c^	116
17	D	0	2	24	11	30	3.5 ^b^	94
5	MV	0	1	20	19	45	3.9 ^b^	1076
			mean	52	32	73	8.3	330
			median	47	28	57	3.9	248
	Total symptom score	*r* (*p*) ^e^	0.53 (0.04)		0.97 (<0.001)	0.44 (0.10)	−0.12 (0.67)	−0.06 (0.83)
	MD symptom score		0.47 (0.08)			0.47 (0.08)	−0.19 (0.50)	−0.01 (0.97)
	MRI to urine Gd						−0.49 (0.06)	0.08 (0.77)
	24 h urine Gd level							−0.18 (0.53)

^a^ Linear: MH—gadobenate dimeglumine/MultiHance^®^; MV—gadopentetate dimeglumine/Magnevist^®^; OM—gadodiamide/Omniscan^®^; OP—gadoversetamide/OptiMARK^®^). All others are macrocyclic: (G—gadobutrol/Gadovist^®^; D—gadoterate/Doterem^®^). Unprovoked limits: ^b^ Mayo Clinic Laboratory ≤ 0.4 µg/24 h; ^c^ Doctor’s Data ≤ 0.6 µg/24 h; ^d^ Genova Diagnostics ≤ 0.019 µg/24 h. ^e^ Pearson’s correlation.

**Table 2:** Participant symptoms at blood draw.

Symptom	No. (%) ^a^
Tingling sensations ^b^	15 (100)
Fatigue ^b^	14 (93)
Cognitive difficulty ^b,c^	13 (87)
Muscle twitching ^b^	13 (73)
Bone pain	13 (87)
Extremity/Joint pain ^c^	13 (80)
New onset frequent headaches ^b,c^	12 (80)
Skin/muscle pain ^b,c^	12 (80)
Dry eyes ^b^	12 (80)
Skin tightening or thickening ^c^	12 (80)
Chest/Abdominal pain ^c^	10 (67)
Decreased visual acuity ^b^	9 (60)
Skin hyperpigmentation ^c^	8 (53)
Eye pain ^b^	8 (53)
Decreased skin sensation ^b^	7 (47)
Decreased bowel/bladder sensation ^b^	5 (33)

^a^ Total participants (*N* = 15). ^b^ Contributes to MD symptom score. ^c^ Provisional GDD diagnostic criterion.

## Data Availability

Full metabolomics datasets presented in this study are available from the corresponding author on request. The data are not publicly available due to restrictions in place for privacy and ethical concerns.
